# Towards Autonomous Robotic Minimally Invasive Ultrasound Scanning and Vessel Reconstruction on Non-Planar Surfaces

**DOI:** 10.3389/frobt.2022.940062

**Published:** 2022-10-11

**Authors:** Nils Marahrens, Bruno Scaglioni, Dominic Jones, Raj Prasad, Chandra Shekhar Biyani, Pietro Valdastri

**Affiliations:** ^1^ Storm Lab UK, Institute for Robotics, Autonomous Systems and Sensing, School of Electronic and Electrical Engineering, University of Leeds, Leeds, United Kingdom; ^2^ Department for Hepatobiliary Surgery, James’s University Hospital, Leeds Teachings Hospitals NHS Trust, Leeds, United Kingdom; ^3^ Department for Urology, James’s University Hospital, Leeds Teachings Hospitals NHS Trust, Leeds, United Kingdom

**Keywords:** autonomous robotic ultrasound, robotic surgery, vessel reconstruction, tissue coupling estimation, non-planar scan surface, anatomy based navigation

## Abstract

Autonomous robotic Ultrasound (US) scanning has been the subject of research for more than 2 decades. However, little work has been done to apply this concept into a minimally invasive setting, in which accurate force sensing is generally not available and robot kinematics are unreliable due to the tendon-driven, compliant robot structure. As a result, the adequate orientation of the probe towards the tissue surface remains unknown and the anatomy reconstructed from scan may become highly inaccurate. In this work we present solutions to both of these challenges: an attitude sensor fusion scheme for improved kinematic sensing and a visual, deep learning based algorithm to establish and maintain contact between the organ surface and the US probe. We further introduce a novel scheme to estimate and orient the probe perpendicular to the center line of a vascular structure. Our approach enables, for the first time, to autonomously scan across a non-planar surface and navigate along an anatomical structure with a robotically guided minimally invasive US probe. Our experiments on a vessel phantom with a convex surface confirm a significant improvement of the reconstructed curved vessel geometry, with our approach strongly reducing the mean positional error and variance. In the future, our approach could help identify vascular structures more effectively and help pave the way towards semi-autonomous assistance during partial hepatectomy and the potential to reduce procedure length and complication rates.

## 1 Introduction

Despite the advances in other areas such as dexterity and image quality, commercial surgical robotic systems lack autonomy and are merely used as tools for the teleoperation of surgical instruments (Attanasio et al., 2021). Autonomy in surgical robotics could help solve a plethora of issues, such as the shortage of medical staff, availability of adequately trained surgeons, and could potentially enable them to perform other, more relevant tasks or surgical procedures instead. A critical point in enabling this is the knowledge of the patient’s specific anatomy to adequately define resection margins for extracting diseased tissues and safely manipulate anatomical structures. While pre-operative imaging can help gain useful insights, the exact mapping of these images to the surgical site is unknown under general circumstances, both to the human or the computer. This particularly applies to highly flexible organs such as the liver, where the positioning of the patient during surgery along with *C O*_2_ insufflation may cause significant changes in position and shape of the organ (Zhang et al., 2021). In this and other surgical scenarios, intraoperative US scanning is often adopted to acquire knowledge of the anatomy (Zhu et al., 2018).

While many research efforts have been dedicated to exploring automated scanning on the patient’s skin (extracorporeal US - Pierrot et al. (1999); Elek et al. (2017)), scanning inside the patient during minimally invasive surgery (intracorporeal US - Pratt et al. (2015); Schneider et al. (2016)) poses several unaddressed challenges. Extracorporeal US systems adopt serial manipulators that greatly simplify precise and reliable spatial movements and force measurements. An extensive review on robotic systems for manipulation of extracorporeal US may be found in Elek et al. (2017). Research work includes both systems for teleoperation, often integrating force feedback (Pierrot et al., 1999), visual servoing methods (Abolmaesumi et al., 2002; Royer et al., 2015), as well as more recently the creation of autonomous systems in Xiao and Wang (2021) and Jiang et al. (2021). In the context of improving robotically assisted surgery, prior works mostly use extracorporeal (Mathur et al., 2019) or endolumninal probes (inserted through natural orifices), particularly Transrectal Ultrasound (TRUS), controlled via an external robot (Hung et al., 2012; Mohareri et al., 2012) to visually track robotic instruments and target regions. Due to their increased distance to the operating region, extracorporeal probes lack the details of a more close-up scan of the surgical site under general circumstances. Endoluminal US on the other hand, is restricted to target regions that lie close to orifices (e.g., prostate) or luminal organs (e.g., esophagus) accessed via flexible endoscopes.

Minimally invasive surgical systems, on the contrary, are built compliantly, commonly with tendon-driven instruments as their end-effectors, making both precise kinematics and force measurements inherently difficult (Nia Kosari et al., 2013). Moreover, the visual information acquired through US must be spatially mapped to the guiding robot for it to be meaningfully applied in an assistive or autonomous application. To this end, previous research involving surgical robots mainly resorted to optically tracking instruments and probe (Pratt et al., 2015). This is a feasible approach in the presence of a stereo endoscope, but lens distortion and the short disparity between the two lenses of the endoscope reduces its accuracy and applicability, and the markers need to be visible in the scene, posing a set of very restrictive assumptions. The use of alternative technologies such as electromagnetic tracking are limited by the presence of metallic instruments (Schneider et al., 2016) or metallic elements in the surgical table and the potential disruption to the surgical workflow, requiring setting up and placing the field generator near the surgical site on the patient. Various works have treated the development of hardware devices to facilitate Robotic Intracorporeal Ultrasound (RICUS) (Schneider et al., 2016) or methods for registering robotic instruments with RICUS probes (Mohareri and Salcudean, 2014). Recent work outlined in Stilli et al. (2019) reports a novel rail mechanism that allows the US probe to be spatially fixed to the scan location, thus improving scan stability and facilitating spatial registration of the scan. While the results are encouraging, the device may limit the possible movements of the probe or block access to sites of interest compared to a scan purely guided by a robotic instrument. Different tracking methods (kinematics, optical tracking and electronic-magnetic tracking) for theda Vinci Research Kit (dVRK) Patient Side Manipulator (PSM)’s position are compared in Schneider et al. (2016) to reconstruct a vessel phantom. The authors report a target registration error of 5.4 ± 1.7 *mm* using the dVRK kinematics. Pratt et al. (2015) describes a system for autonomous tissue resection. The work focuses on tracking and autonomously cutting along a line rather than navigating along anatomical features. Moreover, the work employs external visual trackers, a very restrictive assumption that may limit the functionality in a realistic clinical environment.

None of the references on RICUS give clear indications as to how they ensured adequate coupling between the US probe and tissue surface. Pratt et al. (2015) demonstrates scans on a planar surface, not needing to reorient the probe after an initial set orientation (Pratt et al., 2015). Schneider et al. (2016) describes scanning a non-planar surface, presumably telemanipulated rather than autonomously. In extracorporeal US coupling may be ensured via 3D perception of the environment in combination with a force torque sensor within the robotic end-effector or directly inferred from the join torques. These methods, however, are not easily transferable to a minimally invasive setting, where sensor size, robot inaccuracies and the visual complexity of the scene limit the practical application. Another approach is to use an ultrasound image-based solution, such as ultrasound confidence maps (Karamalis et al., 2012), in combination with a force sensor (Chatelain et al., 2017).

In this work, we follow a similar approach, based, however, on a simplified, easily scalable, data-driven algorithm to estimate the local tissue coupling quality. Building upon the works of Jiang et al. (2021) and Xiao and Wang (2021), we extend the idea of autonomous US scanning along anatomical features to a minimally invasive surgical robot and intracorporeal US As a core part of this endeavour, we present and evaluate a method for fusingg Inertial Measurement Unit (IMU) data with robot kinematics. In doing so, we improve the robot’s localization in 3D space and enable a more precise and reliable anatomic reconstruction from the US scans, despite the robot’s kinematic inaccuracies and without the need for restricting tracking systems. In summary, this work presents an approach that is easily scalable to multiple vessels, can deal with the absence of reliable force sensing and does not require external spatial tracking. We claim that our aforementioned contributions provide a first step towards enabling more reliable autonomous intracorporeal ultrasound scanning, with a strong focus on providing a solution that is easily adaptable to a clinically realistic scenario and workflow. We use and assess our approach on a dVRK (Kazanzides et al., 2014), the standard platform in research on robotic minimally invasive surgery.

## 2 Materials and Methods

**FIGURE 1:**
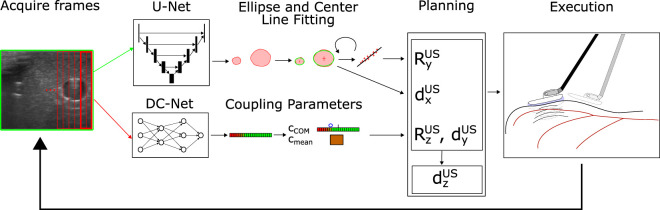
System Structure: The current US frame is acquired and fed into U-Net (entire image) and DC-Net (overlapping image slices). The segmentation results of the U-Net are post-processed, and ellipses are fitted around segmentation patches likely to correspond to vessels (upper path). The current vessel center is used to perform in-plane movements (*d*_*x*_) to center the vessel within the image. Recognized vessel centers from previous frames are buffered and used to estimate the center line and reorient the image normal $R_{y}^{US}$ (rotation around *y*_*US*_ - see also Figure 3). The parallel path with the outputs from the DC-Net are compressed into coupling parameters that are used to adapt rotation around the image normal (*R*_*z*_) and compression movements (*d*_*y*_). If the probe alignment with the vessel and coupling is properly adjusted, the probe is progressed forward in the image plane normal direction (*d*_*z*_).

Our considered setup consists of a single PSM (also referred to as robot) of a dVRK (Kazanzides et al., 2014) and a Philips L15-7io probe driven by an iU22 US machine (Philips, Amsterdam, NL). The dVRK enables the control of a formerly clincal da Vinci PSM and implements ROS middleware to directly set target values for the robot servo control loop. It also includes inverse kinematics to allow the specification of target values in Cartesian space. The PSMs are designed to rotate an integrated trocar tube around a fixed fulcrum, kinematically restricting movements and mechanical stresses around the incision point. We use the currently newest version (dVRK ROS 2.1) including the recently updated re-calibration of the prismatic joint. We chose the imaging specifications of the US probe to be identical to those used in commercially available surgical robotic US probes (7–15 MHz) to make the results and conclusions directly transferable to conventional RICUS In its original form the US probe is intended to be hand-held during open surgery. To adapt it to the PSM’s instruments, we designed a custom 3D printed interface similar to Schneider et al. (2016), allowing it to be compatible with the dVRK’s Fenestrated Bipolar Forceps for stable and repeatable grasping. We further integrate an IMU into the US probe for improved localization despite potentially slightly varying grasps of the probe, as well as a Force Sensitive Resistor (FSR) to prevent excessive normal forces. The pick-up interface further allows the addition of an IR tracking frame to acquire ground truth data for evaluation. For the case of automated vessel scanning, we separate the different spatial degrees of freedom of the US probe into two different categories: anatomy-based and tissue-coupling-based movement adaptions. Anatomy-based movements are made to follow and orient the probe towards the overall anatomic structure, while tissue-coupling-based movement adaptions are primarily due to the scanning surface and the contact with the US probe. Our resulting control and planning scheme follows this logic by involving dedicated image processing pipelines for each category (see Figure 1). In the planner, both branches converge to decide on the most urgent task to be performed: coupling adaption ( $R_{z}^{US}$ , $d_{y}^{US}$ ), vessel centering $\left(d_{x}^{US}\right)$ , vessel center line alignment $\left(R_{y}^{US}\right)$ , and probe forward progression $\left(d_{z}^{US}\right)$ (see more detailed outline in Section 2.5).

### 2.1 Nomenclature

In the upcoming, sections we will make use of mathematical notation, to denote the several coordinate frames and transformations between those frames defined in our system. Transformations with a mere subscript such as *T*_*A*_ (including *R*_*A*_ and ${\vec{p}}_{A}$ ) are used to refer to the coordinate frame itself, while the transformation from a coordinate frame *T*_*B*_ to coordinate frame *T*_*A*_ is written as $T_{A}^{B}$ and denoted with subscript and superscript. Additionally, we use comma-separated superscripts such as $T_{A}^{B,des}$ to specify the relative poses, in this case the desired (abbreviated as *des*) relative pose of *B* with respect to A. Expressed in other terms, $T_{A}^{B}$ is the pose of *T*_*B*_ relative to *T*_*A*_. Broken down further, $T_{A}^{B}$ is defined as a homogeneous transformation, composed of a rotation $R_{A}^{B}$ and a translation ${\vec{p}}_{A}^{B}$

$$T_{A}^{B}=[\begin{array}{ccc} R_{A}^{B} & \; & {\vec{p}}_{A}^{B}\\ 0\;0\;0 & 1 \end{array}]$$ (1)

In vectors, we use uppercase letters for the superscript to denote defined coordinate frame quantities (e.g., relative position of the origin ${\vec{p}}_{A}^{B}$ or coordinate axes ${\vec{x}}_{A}^{B}$ , ${\vec{y}}_{A}^{B}$ and ${\vec{z}}_{A}^{B}$ ). We further use lowercase letters to specify relative positions without defined coordinate frames. An example of this is the changing center line of the vessel $t_{A}^{cl}$ . The only exception to this is the gravity vector *g*_*A*_, which does not specify a reference frame or lowercase subscript, as gravity is a world-implicit quantity (e.g., implying a specific orientation).

### 2.2 Inertial Measurement Unit

Initial experiments highlighted that the dVRK’s instruments have pronounced backlash, particularly in the last three joints at the tip of the instrument. Even without external loads, the orientation of the instrument is not accurately determined, which is further amplified along the length of the end-effector link towards the origin of the US base frame *T*_*US*_. Tendon-driven robots are known to have such drawbacks that increase over time and usage and result in that add up along the chain of links (Nia Kosari et al., 2013). This effect is more pronounced with external loads at the instrument tip. US scanning involves two different sources of external loads. Firstly, loads induced by the probe itself due to its weight (force), weight distribution (torque), and its cable (force and torque). Secondly, the interaction between probe and tissues creates a force normal to the contact surface, as well as friction forces within the contact plane. The sum of forces induced by the probe and the contact result in significant variations of the probe’s actual position and orientation that is not measured by the PSM’s joint encoders.

**FIGURE 2:**
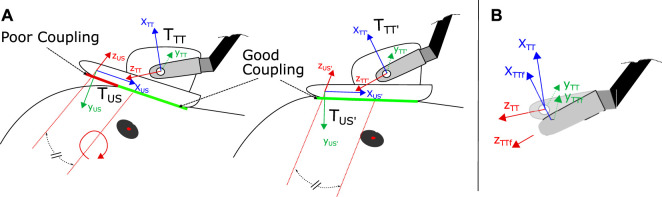
**(A)** Coupling adaption of the US probe via rotation around *z*_*US*_ axis (equivalent to image normal vector), shifted into the center of the image probe. To determine the amount and direction of probe angle adaption we use the condensed *c*_*COM*_ parameter (see Eq. 3) **(B)** Update of the end-effector orientation and global position *T*_*TT*_ via attitude sensor fusion resulting in fused tool tip frame *T*_*TTf*_. Based on *T*_*TTf*_, *T*_*US*_ is updated as well (not depicted for simplicity).

To compensate for these effects and to improve the kinematic estimation, we integrate an IMU (Bosch BNO055, Bosch Bosch GmbH, Stuttgart, Germany) measuring gravity and thus the orientation of the global *T*_*PSM*_. We ignore the integrated magnetometer readings, considering it an unrealistic assumption for a future application in a clinical setting. We further ignore the measured accelerations and rotational velocity readings, as these measurements were found to be too noisy under the slow movements our system performs. To fuse the available information between the robot kinematics and the IMU, we consider the spatial orientation determined by the robot kinematics as well as the gravity vector from the IMU, transformed into *T*_*US*_. Following the logic of a Mahony filter (Mahony et al., 2008), we then calculate the update in orientation in the following way:

$$\Delta {\vec{\omega }}_{PSM}^{US}={\vec{z}}_{PSM}^{US}\times \frac{{\vec{g}}_{US}}{\left\|{\vec{g}}_{US}\right\|}$$ (2)

where ${\vec{z}}_{US}^{PSM}$ is the *z*-axis of the global frame *T*_*PSM*_, expressed in *T*_*US*_, and ${\vec{g}}_{US}$ , the direction of gravity expressed in *T*_*US*_. Employing the quaternion product, we map $\Delta \omega_{PSM}^{US}$ into a quaternion velocity and update $T_{PSM}^{US}$ . We then go back in the kinematic chain toward the end-effector link to the ultimate joint and update the end-effector location based on the updated orientation (see Figure 2B). Unlike the Mahony filter, we apply the full update in each step $\left(K_{p}=\frac{1}{\Delta t}\right)$ and break the recursiveness by newly starting with the measured ${\vec{z}}_{PSM}^{US}$ from the robot’s kinematics in each time step. In the following, we will refer to this approach as PSM-IMU-fused kinematics, as opposed to the pure PSM kinematics obtained from joint encoder reading and forward kinematics.

### 2.3 Vessel Segmentation

For Vessel segmentation, we use a U-Net (Ronneberger et al., 2015), in line with previous works such as Jiang et al. (2021) and Xiao and Wang (2021). The network is trained with manually collected and labeled data from two anatomically different US vessel phantoms. Our loss function is a combination of binary-cross entropy and dice loss. Contained in the dataset are a total of 697 labeled images (non-augmented), splitting up into 592 (85%) for training and 105 (15%) for evaluation. Each image contains up to three vessel cross-section labels, with the majority containing a single one. Our final model has a validation Dice score of 0.887, which is lower than the 0.982 reported in Jiang et al. (2021). We attribute this to the data augmentation, specifically image color inversion (roughly ten percent of the images) that we apply. While the techniques lead to more robust results, they also result in very inaccurate predictions for these specific samples. For training and inference, we employed an NVIDIA Quadro RTX5000. The final segmentation during our experiments runs at speeds of 7-8fps. This is prior to further performance improvements of our code, which are expected to further boost the frame rate.

**FIGURE 3:**
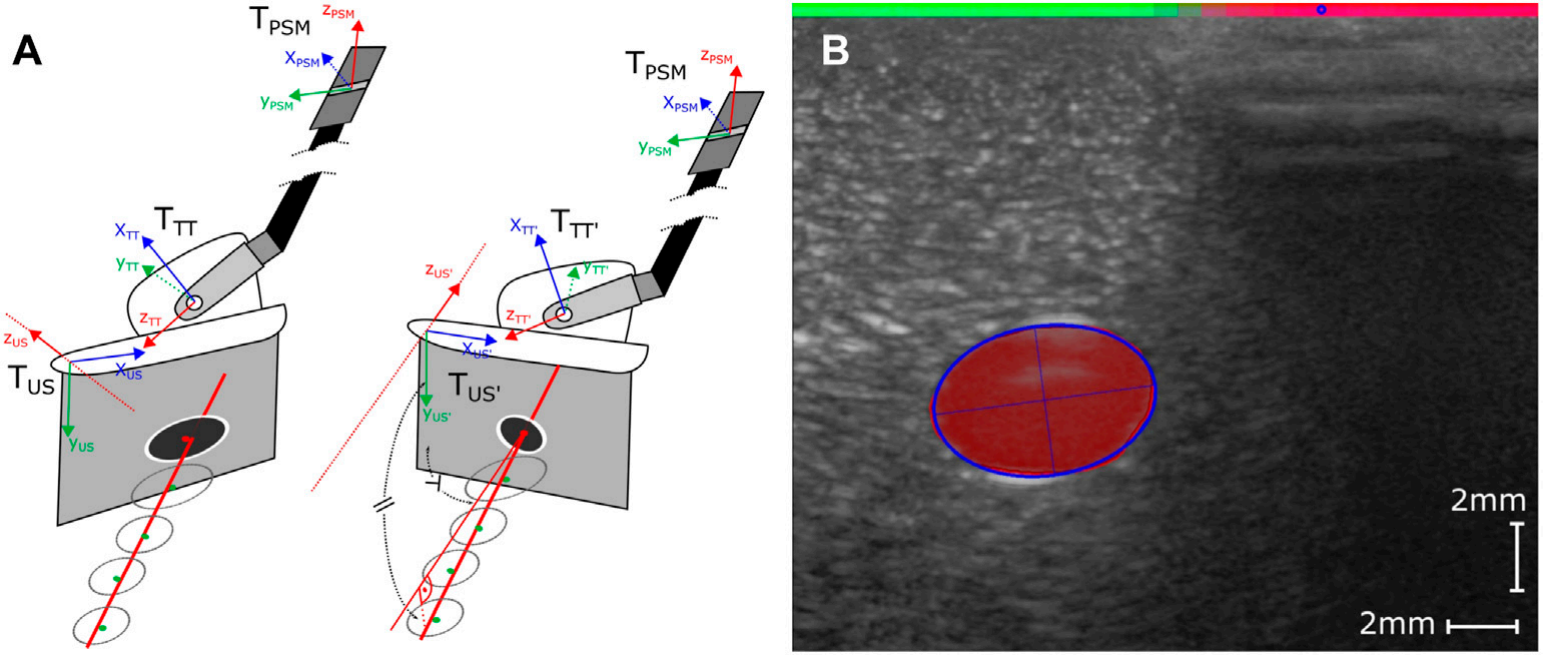
**(A)** Considered coordinate Frames *T*_*US*_, *T*_*TT*_ and *T*_*PSM*_ Left: Before adaption of the image plane normal (poor orientation *T*_*US*_ and *T*_*TT*_), Right: After adaption of image plane normal (ideal orientation), based on projected vessel center line (good orientation *T*_*US*’_ and *T*_*TT*’_). **(B)** Visual representation of the post-processed outputs of the two Deep Neural Networks. The fitted ellipse (blue outline) closely tracks the segmentation results of the U-Net (area in red). The detected coupling quality by the DC-Net is visualized by the colored bar on the top transitioning from green (good coupling) to red (poor coupling) and the blue circle representing the calculated *c*_*COM*_ parameter.

In its raw form, the U-Net represents segmentation masks as binary pixelated images. Further processing is therefore needed to compress the data into more meaningful vessel features. Therefore, we extract the contours from the filtered image and fit ellipses around the contours using OpenCV, allowing a differentiation between several vessel instances (instance segmentation). By assuming a lower threshold vessel diameter of 20 pixels (corresponding to a vessel diameter of around 0.45 *mm*) for the navigational task at hand, we can further clean up potential erroneous or irrelevant vessel detections. Figure 3B shows a typical result of a detected vessel after post-processing the segmentation. We start by applying some initial filtering (erosion, dilation, thresholding) to eliminate small noisy patches that are unlikely to correspond to actual vessels. Unlike the approach of resampling the vessel followed by Jiang et al. (2021), this approach allows the differentiation between various vessel instances or bifurcation points present in the image. While during this proof of concept study, we consider only a single vessel, addressing bifurcations, could be interesting work for the future.

### 2.4 Visual Coupling Quality Estimation

For quantification of the coupling quality and the detection of a decoupled probe, we propose a convolutional neural network, in the following referred to as Decoupling Network (DC-Net). Due to the wave-nature of US a poor coupling between the probe and tissue in a location on the sensor will affect the whole image slice along the propagation direction of the US wave (depth of the image). Rather than processing an entire image at once and solving the regression task of estimating the coupling quality across the image’s width, we propose feeding the network fully overlapping slices, including half of the information from each neighboring slice. This vastly reduces the size of the network and simplifies it to a binary classification task (good/poor coupling) on each separate slice. This approach additionally increases the number of available data samples for training and evaluation. Making the slices overlap increases the robustness of the system due to the inclusion of partly redundant visual information. Each image is split into 32 slices, which we chose to be a compromise between resolution and performance.

**FIGURE 4:**
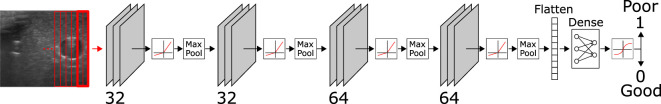
Structure of the DC-Net with four convolutional layers, each directly followed by a leaky ReLu layer and anisotropic max pooling. The flattened output of the first four layers is further processed through two dense layers, including an in-between ReLu and dropout layer (*p* = 0.5) and a final softmax layer for binary classification into coupled (0) and decoupled (1).

The network is comprised of four convolutional layers with depths 32, 32, 64, and 64, respectively, followed by two dense layers. Between each of the convolutional layers, we apply a leaky ReLu activation (*alpha* = 0.05) function followed by anisotropic max pooling (4 in depth and 2 in width dimension). The latter accounts for the large pixel ratio of 8:1 between the depth and width of the extracted slices. Following the convolutional layers, we process the flattened output through two dense layers with an in-between ReLu activation function and dropout of 0.5. The final classification result is generated via applying a softmax activation to the output of the last dense layer (see Figure 4), resulting in values between 0 (coupled) and 1 (decoupled). The data set is comprised of a total of 6,634 image slices with 40% of samples labeled poorly coupled and 60% well coupled. After 250 training epochs on 85% of data samples, we reach an overall validation accuracy of 0.99 on the remaining 15% of samples.To reduce the number of classified slices into parameters that give meaningful indications for probe adaptions, we propose two parameters that summarize the coupling over the width of the image and are used to adjust the probe orientation and position. We first calculate the center-of-mass (CoM) equivalent of the classified coupling quality as follows

$$c_{COM}=\frac{\sum_{i=0}^{n}\left(c_{i}d_{i}\right)}{\sum_{i=0}^{n}d_{i}}$$ (3)

with *n* being the number of slices in the image, *c*_*i*_ the coupling quality of slice i, and *d*_*i*_ the distance of the center of the slice to the center of the image. We train the network to classify slice as strictly 0 or 1; however we use the floating-point values given by the ultimate softmax layer to result in values between 0.0 (good coupling) and 1.0 (poor coupling) for our further calculation of coupling quality parameters. While the values might seem counter-intuitive at first glance (higher value equals lower coupling quality), they were chosen to represent 0 as the default state (good coupling) compared to in-equilibrium as any offset from 0 (poor coupling). If all slices are near 0 (e.g., smaller than 0.1), we expect the *c*_*COM*_ to be in or very close to the center of the image.

### 2.5 Planning and Control

**FIGURE 5:**
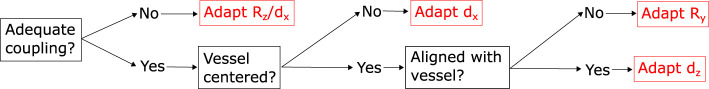
Outline of the hierarchical planning routine, employing different tracks for adapting the probe orientation and position with respect to the tissue surface or the reconstructed anatomy. We prioritise in the order of coupling over the centering of the vessel and finally the alignment of the vessel center line.

To simplify the control problem, we split the probe manipulation problem up into four independent movements (see Figures 3, 5): orientation matching with the projected center line of the vessel (rotation around *y*_*US*_-axis), vessel centering (in-plane movement in *x*_*US*_-direction), forward movement along the vessel (out-of-plane movement in *z*_*US*_-direction) and coupling optimization (in-plane movement in *y*-direction and rotation around *x*_*US*_-axis). The only direction not considered for adaption is rotation around *x*_*US*_, which for the purposes of this study we assume to remain nearly constant. Each control cycle starts with a check of the coupling quality; further movements are considered in case of good coupling quality. Otherwise, the coupling is adjusted until reaching an acceptable level. We found this to be |*c*_*COM*_ < 0.1|. This is to ensure that the feature is not suddenly lost due to the poor coupling of the probe. Assuming the coupling is adequate, the planner continues with a proportional controller to keep the detected vessel centered in the image frame. In the subsequent layer, the planner checks for deviations of the image plane normal with respect to the estimated vessel center line projection (see Figure 3). If no orientation adaptions are needed, meaning the probe is well coupled, with the vessel centered and the image plane normal *z*_*US*_ orientated in accordance with the current center line estimate, the probe is propagated forward in image plane normal direction (in direction of *z*_*US*_). All routines further include a limited proportional controller to keep the vessel centered in the image.

If one of the sides of the probe decouples, we expect *c*_*COM*_ to shift toward the decoupled side (see Figure 3). As a result, we trigger the planner to tilt towards the respective direction to re-establish coupling. This process is depicted in Figure 2A, where the probe is rotated around *z*_*US*_ shifted into the middle of the probe (half a probe width into *x*_*US*_ direction). In addition to *c*_*COM*_ we consider the overall mean as well as the mean of the left, central and right third of the image to allow downwards probe movements in the case that the entire probe is not well coupled.

**FIGURE 6:**
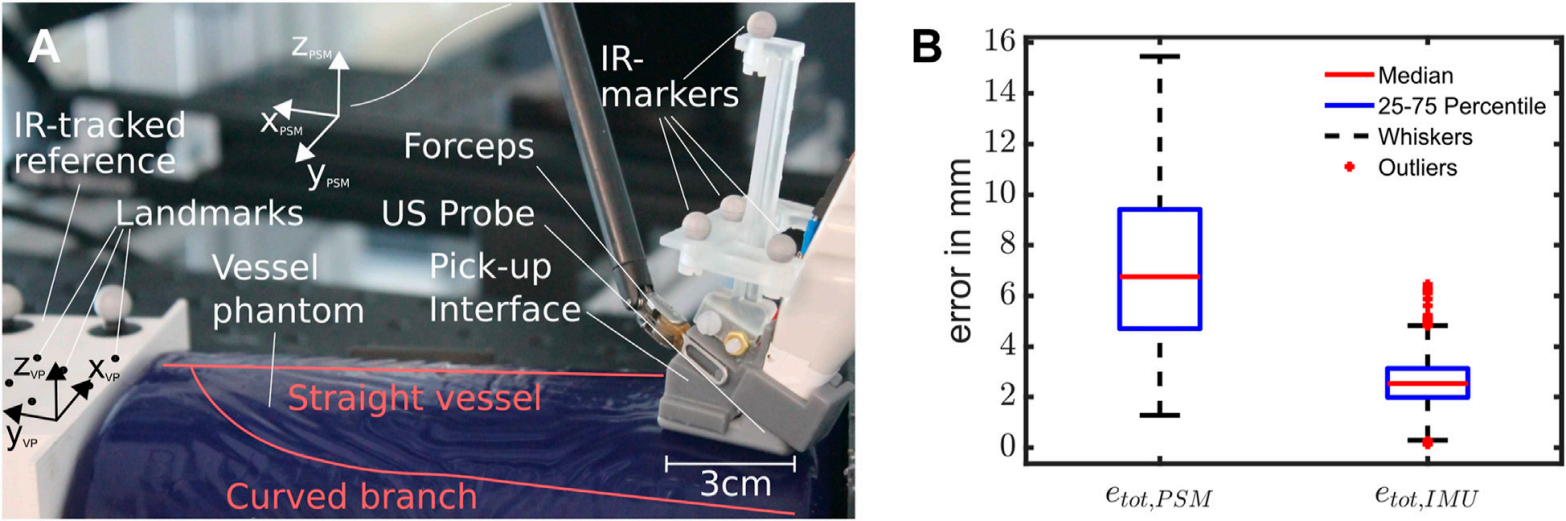
**(A)** Setup for the experiments, with a roughly overlayed outline of the vessel structure inside the phantom, the ultrasound pick-up probe with integrated IMU and FSR and the reference block. **(B)** Box Plot showing the error distributions of the resulting scans using pure PSM- or IMU-based kinematics. Outliers are considered to be values outside the 1.5-fold interquartile range from each side of the box, demarked by whiskers (*n* = 12,596 data points from ten scans).

In order to prevent applying excessive pressure to the tissue, we integrate a simple force sensitive resistor into the setup (see Figure 6A). The sensor is located between the pick-up interface and top of the US probe. Any normal force applied to the probe via the robot will travel through this element and thus register any normal force applied to the probe. We calibrated the integrated force sensor by fixating the probe on its pick up interface and applying successively increased loads. FSR may not give very accurate force reading and tend to drift over time. Therefore, we only use the sensor to prevent excessive forces from being applied. Drifting may be prevented by zeroing the probe before each scan, e.g., while holding it slightly above the tissue surface to be scanned. This process may even be automated in the future. In our current setup, we set the force not to exceed 3.5*N*, which includes the weight of the probe, so the contact force with the tissue will be far below this value. In case 3.5*N* are exceeded, we lift the probe off in direction of *y*_*US*_ until we are below the threshold to the optimise potential further decoupling on the sides of the probe. Furthermore, we found this to be the optimal value for ensuring good coupling on the given phantom, while preventing noticeable deformations. This value may differ for different probes, softer phantoms or real tissue. While this extra sensor does constitute additional integration effort, we believe they are far easier to be integrated than a full three or even six degrees of freedom force sensor.

A further assumption we are making is that sufficient liquid is present on the scanning surface. As opposed to the patient’s skin the inside of the abdominal cavity is sufficiently moist, and additional water is usually added via an irrigation rod prior to scanning. In order to adapt out-of-plane movements with respect to the vessel, we consider the last 35 detected vessel center points in globally fixed PSM coordinates. We filter the point cloud to be only valid if most samples include a detected vessel, no major probe decoupling occurred, and the image normal plane remained constant. Furthermore, only movements in the estimated normal direction will produce point clouds that contain valid information about the actual current orientation of the vessel’s center line. This ensures that the slope can actually be estimated. We encode this by enforcing that 89% of the last 30 samples are valid, which we empirically determined to be a good value for stable estimation of the center line. If this criterion is not fulfilled, we keep the current orientation as the best estimate. Thereby, we minimise the potentially high amounts of noise from the kinematics during rotational movements to affect the estimated center line orientation and thus the image normal adaption. Additionally, we impose a minimal distance covered between the points in the current point cloud to ensure that a line fitting is feasible and accurate. While we ignore the point cloud sets obtained from non-normal scan movements, we still include them for the final reconstruction of the vessel. We determine the center line slope via a separate least-square line fitting for each dimension. We transform the resulting slope into the US probe frame and project the center line onto the *x*_*US*_-*z*_*US*_-plane (see right of Figure 3A). This ensures that the probe only rotates around assumed surface normal *y*_*US*_ and does not lift off or push further into the surface, altering the coupling in unexpected ways. The approach can be summarized with the following formula:

$${\vec{z}}_{PSM}^{US,des}=R_{PSM}^{US}\cdot \frac{I_{xz}\cdot {\left(R_{PSM}^{US}\right)}^{-1}\cdot {\vec{t}}_{PSM}^{cl}}{\left\|I_{xz}\cdot {\left(R_{PSM}^{US}\right)}^{-1}\cdot {\vec{t}}_{PSM}^{cl}\right\|}$$ (4)

$$I_{xz}=\left(\begin{array}{ccc} 1 & 0 & 0\\ 0 & 0 & 0\\ 0 & 0 & 1 \end{array}\right)$$ (5)

where ${\vec{z}}_{PSM}^{US,des}$ is the new desired orientation of the *z*_*US*_-axis expressed with the PSM coordinate frame *T*_*PSM*_, $R_{PSM}^{US}$ is the rotation component of the homogeneous transformation between PSM and US probe coordinates, *I*_*xz*_ a selector matrix to filter out the *y*-component in the US probe’s coordinate frame, as we are rotating around this axis, and ${\vec{t}}_{PSM}^{cl}$ the orientation vector of the fitted center line. To set up a consistent right-hand coordinate frame, the remaining *x*_*US*_-axis in PSM coordinates is determined as

$${\vec{x}}_{PSM}^{US,des}={\vec{y}}_{PSM}^{US}\times {\vec{z}}_{PSM}^{US,des}$$ (6)

where *y*_*PSM*_ is the current *y*-component of the Rotation matrix $R_{PSM}^{US}$ that describes the *y*-axis of the US probe coordinate frame in PSM coordinates. The final desired probe rotation $R_{PSM}^{US,des}$ is built by combining all axes

$$R_{PSM}^{US,des}=\left(\begin{array}{ccc} {\vec{x}}_{PSM}^{US,des}, & {\vec{y}}_{PSM}^{US,des}, & {\vec{z}}_{PSM}^{US,des} \end{array}\right)$$ (7)

## 3 Experimental Validation

In setting up the system, we determined the transformation between the US image and the robotic end-effector. Using the metric correspondence given by the manufacturer, we converted the image to a metric scale. To acquire the positional offset and a more precise estimate for the transformation between the PSM and the US image of the probe, we built a custom cross-wire phantom. To reference the scan, we used an Infrared (IR) optical tracking system consisting of four Optitrack Primex 13 (NaturalPoint, Inc., Corvallis, OR, USA) with markers attached to the probe as well as the calibration phantom. We obtain the final transformation via iterative closest point (ICP) matching.

To mimic the setting of a hepatectomy, we base our experiments around a BLUEPHANTOM Branched 2 Vessel US Training Block Model (CAE Healthcare, Saint-Laurent, Quebec, CA), depicted in Figure 6A. We chose the vessel phantom to resemble vessels similar to those found in the liver (diameter: 4 − 6 *mm*). The phantom consists of a straight main vessel with a second vessel branching off around 45 *mm* down the length of the vessel at a 60°–70° angle. This vessel slowly curves until it runs roughly parallel to the straight vessel (see Figure 6A), finally bending downwards at a roughly 15°. Since we do not know the exact vessel location and orientation in 3D space, we added an IR tracking system to the experimental setup that is used to acquire ground truth data (accuracy of 0.1 *mm*) however is not intended as part of the setup used during a potential future surgical application. To acquire a mapping between the IR tracking system and the PSM end effector, we use a 3D printed frame that is tracked via the IR tracking system. On the side of the PSM we spatially register the frame to the *T*_*PSM*_ via touching spatial landmarks and calculating the resulting transformation. All robotic and US data, including the segmentation results and extracted vessel ellipses, are published on ROS topics for further processing and exchange between the several program routines.

Our experiments are comprised of scans of the curved vessel as a non-trivial geometry for probe orientation. The scan starts from the vessel’s straight section (roughly parallel to *y*_*VP*_ on one end of the phantom), over towards its turn, and until it merges with the main vessel towards the other end of the phantom. Due to the turn and the varying starting angles, it is practically impossible to fully scan the vessel without stable probe orientation adaption that ensures an image normal approximately parallel to the projected center line of the vessel. For each scan, we start in close proximity to one end of the phantom with the probe partly decoupled from the tissue surface (similar to Figure 3) and with an image normal orientation clearly deviating from the center line of the vessel. The first five scans started with a rotational deviation of the center line turned towards one side, while the other five were started with a rotational deviation towards the opposite side. During the scans, we recorded the robot’s position along with the detected vessel centers and axes. All scans in the evaluation are extracted from runs using IMU-PSM-fused kinematics for control (see accompanying video for an exemplary scan).

To assess the resulting scans and compare them, we calculate the root squared error (euclidean distance) between the reconstructed vessel center points using IR tracking with that of pure PSM or PSM-IMU-fused kinematics. We reduce our analysis to the estimated center points, as the detected radius of the vessel will be the same for all methods and does not add any comparative meaning between the different methods. Furthermore, we calculate the mean difference in orientation between the IR tracking and both kinematic methods to validate and quantify the kinematic improvements made by the addition of the IMU.

## 4 Results

Over ten runs, we observed a mean error of 7.19 ± 6.24 *mm* for pure PSM kinematics and 2.58 ± 1.70 *mm* for PSM-IMU-fused kinematics (both with *p* = 0.05). The maximum errors observed were 15.45 and 6.45 *mm* for PSM and PSM-IMU-fused kinematics, respectively. The medians and quantiles of the error that are depicted in the box plot in Figure 6B are 6.76 *mm* (median), 4.71 *mm* (25% quantile) and 9.4 *mm* (75% quantile) for PSM kinematics and 2.57 *mm* (median), 1.98 *mm* (25% quantile)/3.1 *mm* (75% quantile) for PSM-IMU-fused kinematics. The plot includes zero outliers for PSM-based kinematics and 66 outliers for PSM-IMU-fused kinematics from a total data 12,596 data points.

**FIGURE 7:**
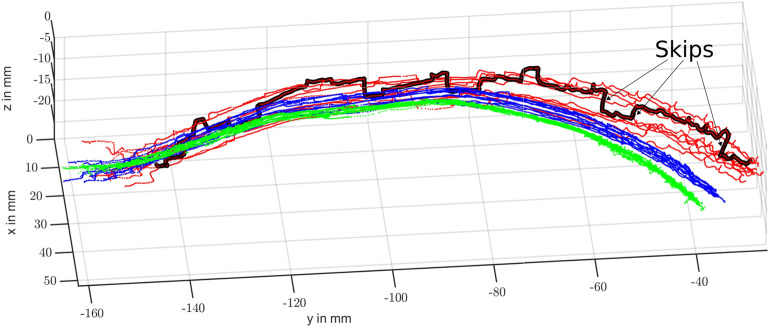
Resulting scans of the curved vessel branch using pure PSM-based (red), IMU-PSM-fused (blue) and IR tracked (green) kinematics. One of the PSM kinematic-based scans with particularly large skips is highlighted in black.

Comparing the trajectories depicted in Figure 7, we observe that all scans obtained via PSM-IMU-fused kinematics are closer to the true scans obtained via IR tracking. Trajectories obtained with pure PSM kinematics can also be observed to end at largely varying positions and heights (*z*_*V*_ *P*). This is not the case for PSM-IMU-fused kinematics. This may be largely explained by the play in the joints and the compliant structure that is not accounted for in the PSMs kinematics. Secondly, the applied correction of the tool tip position in the PSM-IMU-fused kinematics is able to substantially reduce skips and positional drifts, which are strongly pronounced in the trajectories for pure PSM kinematics (see highlighted trajectory in Figure 7). The skips are most likely caused by play in the joints. They express the most during probe orientation of adaption, which strongly involves the two joints with the longest tendons, located near the end-effector. While the robot assumes it is moving and changing its end-effector orientation and position, it actually stays static until the joint properly engages and the joint movement is starting to be transmitted along the full kinematic chain. Along with the ten scans we perform using PSM-IMU-fused kinematics, we executed a total of ten scans using pure PSM kinematics for control but found that only four out of the ten scans were completed successfully (reaching the bifurcation point), since the skips caused the vessel center line estimation to drift off.

When we compare our results obtained with pure PSM kinematics with the results reported in Schneider et al. (2016), we register a higher mean error (7.19 *mm* compared to 5.40 *mm*), while observing a vastly larger significance interval (4.52 *mm* compared to 1.7 *mm*). We assume the authors denoted the single standard deviation, in which case our previously given value of 4.52 *mm* would halve to 2.76 *mm*. Still, the larger value could have several causes. Firstly, we performed many rotations of the tip, which is prone to give more inaccurate results and showed to cause a lot of the skips that will increase the error mean, median, and variance. Secondly, due to the geometry of our probe and its attached cable coming out of the top of the handle, our probe will create a larger pull on the end-effector causing more variation. Further potential sources are the difference in tool age and usage, and the difference in robot calibration or play. To quantify the angular error of both methods, without inclusion of the reconstructed vessel, we calculate the angle difference between the obtained coordinate axes for each method and the assumed ground truth, IR-tracked axes. We observe a strong reduction of the mean angular error and its variation from 5.48° ± 4.64° for pure PSM kinematics to 2.60° ± 0.82° (*p* = 0.05) for IMU-fused kinematics when compared with IR-tracked orientation of *z*_*VP*_. For the other axes, which are not directly corrected for by the fusion routine, the mean errors and double standard deviations (*p* = 0.05) stay in a similar range. The results for *y*_*VP*_ and less pronounced also for *x*_*VP*_, show an improvement in mean angular error while simultaneously posing a slightly higher variation (*x*_*VP*_: 5.81° ± 6.13°/5.08° ± 6.97° - *y*_*VP*_: 7.76° ± 4.77°/5.90° ± 5.94°). As we use the same visual information for all three reconstruction methods, including IR-tracking, we also achieve kinematic errors similar to those reported for the reconstruction with 6.27 ± 6.28 *mm* for PSM-based kinematics and 3.17 *mm* ± 1.96 for IMU-fused kinematics.

## 5 Conclusion

Our experiments demonstrate that the proposed system for autonomous intracorporeal US scanning is capable of repeating several scans within the range of a few millimeters. The addition of an IMU proved to be valuable in determining the orientation and position with more stability, showing fewer deviations and less heavy outliers on a straight vessel scan. This is particularly apparent in the steep jumps of the trajectory using PSM-based kinematics, which disappear entirely or appear strongly smoothed with IMU-fused kinematics. Our developed deep learning based method for the detection of probe tissue coupling showed to be robust and useful in adapting and maintaining a well coupled ultrasound image on a convex surface. While the results are encouraging, we are still reliant on the probe being placed in an initial pose in which the vessel is visible in the ultrasound image. Furthermore, we are still using an FSR to limit the force, although we are looking into methods of inferring this information from other sources such as the joint encoders (Wang et al., 2019). A rigorous test of the resulting reliability of these methods is particularly crucial to prevent excessive forces from damaging the tissue. More experiments may also need to be carried out to assess the behaviour under different surface shapes tissue characteristics. These parameters, representing the diverse characteristics found in real tissue, are expected to have an influence on the measured contact force and thus the coupling behaviour between tissue and probe. These in turn are expected to affect the reliability of the presented approach, implying that our current conclusions are for now limited to the presented simplified bench top scenario. From a design standpoint, however, we ensured that our employed technology is straightforward to integrate into a realistic surgical scenario and workflow, employing vision-based solutions and only additions of small sensors with low setup requirements. In its current state, the navigation and planning is limited to a single vessels and disregards potential bifurcations and several vessel cross sections present in the US image, despite the segmentation routine already enabling the distinction between several vessel instances. Our concept study compared both tested kinematics method with an IR-tracked reference assumed to be the ground truth. According to the calibration software the system imposes errors of less than 0.1 *mm* after calibration. Assuming a centered vessel and good contact conditions, we found the mean in-plane detection of the vessel center to be around 1 mm accurate (95-percentile of 1.1 *mm*). For a more in depth evaluation of the reconstruction accuracy, particularly the influence of the image-plane to end-effector registration error, we will need to perform a Computed Tomography (CT) scan of the phantom with integrated fiducial markers that allow for CT-US co-registration (see Schneider et al. (2016)), which was outside of the current scope of this work.

While the system has been designed with the specific application to hepatic surgery in mind, the approach is generally applicable to further robotic surgical procedures involving vessel structures. We believe this information could still be used to create a safe zone around a vessel structure to be spared or help the surgeon to identify further the anatomy (e.g., the relative location to a tumor or liver lobe from pre-operative data). To reach this goal, we will need to look further into reducing sources of kinematic inaccuracies. One way to do this could be the modelling of the compliant elements of the robot, including the backlash, and to extend previous works such as Chrysilla et al. (2019). Additionally, we plan to integrate the distinction between several vessels into the navigation scene, further opening up the possibility to capture the entire vessel tree geometry and integrate pre-operative information for predictive motion planning and navigation. Our eventual goal will be to extend our approach to tissue trials such as *ex-vivo* animals or Thiel-embalmed human cadaver livers.

## Data Availability

The raw data supporting the conclusions of this article will be made available by the authors, without undue reservation.
